# *LPA* Genotypes and Haplotypes Are Associated with Lipoprotein(a) Levels but Not Arterial Wall Properties in Stable Post-Coronary Event Patients with Very High Lipoprotein(a) Levels

**DOI:** 10.3390/jcdd8120181

**Published:** 2021-12-13

**Authors:** Andreja Rehberger Likozar, Aleš Blinc, Katarina Trebušak Podkrajšek, Miran Šebeštjen

**Affiliations:** 1 Department of Vascular Diseases, University Medical Centre Ljubljana, 1000 Ljubljana, Slovenia; andreja.rehbergerlikozar@kclj.si (A.R.L.); ales.blinc@kclj.si (A.B.); 2 Faculty of Medicine, University of Ljubljana, 1000 Ljubljana, Slovenia; katarina.trebusakpodkrajsek@mf.uni-lj.si; 3 University Children’s Hospital, University Medical Centre Ljubljana, 1000 Ljubljana, Slovenia; 4 Department of Cardiology, University Medical Centre Ljubljana, 1000 Ljubljana, Slovenia

**Keywords:** coronary artery disease, lipoprotein(a), rs10455872, rs3798220, KIV-2 repeats

## Abstract

Lipoprotein(a) [Lp(a)] levels are an independent risk factor for coronary artery disease (CAD). Two single-nucleotide polymorphisms (rs10455872, rs3798220) and number of KIV-2 repeats in the gene encoding Lp(a) (*LPA*) are associated with Lp(a) and CAD. Our aim was to investigate whether in patients with stable CAD and high Lp(a) levels these genetic variants are associated with increased Lp(a) and arterial wall properties. Blood samples underwent biochemical and genetic analyses. Ultrasound measurements for the functional and morphological properties of arterial wall were performed. Genotypes of rs10455872 and haplotypes AT and GT showed significant association with Lp(a) levels. Patients with GG showed significantly higher Lp(a) levels compared with those with AG genotype (2180 vs. 1391 mg/L, *p* = 0.045). Patients with no AT haplotype had significantly higher Lp(a) compared to carriers of one AT haplotype (2158 vs. 1478 mg/L, *p* = 0.023) or two AT haplotypes (2158 vs. 1487 mg/L, *p* = 0.044). There were no significant associations with the properties of the arterial wall. Lp(a) levels significantly correlated also with number of KIV-2 repeats (r = −0.601; *p* < 0.0001). In our patients, these two *LPA* polymorphisms and number of KIV-2 repeats are associated with Lp(a), but not arterial wall properties.

## 1. Introduction

Lipoprotein(a) [Lp(a)] has been shown to be an independent risk factor for cardiovascular diseases [1]. Lp(a) is a complex that is composed of low-density lipoprotein (LDL) and high molecular weight glycolipoprotein(a) (ApoA), which are bound together through a double disulphide bond [2]. More than 90% of the variance in Lp(a) levels can be explained by genetic variability of the gene encoding Lp(a) (*LPA*) [3]. Clarke et al. identified two single-nucleotide polymorphisms (SNPs; rs10455872, rs3798220) in the *LPA* gene that were strongly associated with both increased Lp(a) levels and increased risk of coronary diseases [4]. These two SNPs explained 36% of the variation in Lp(a) levels. They also reported that *LPA* variants are carried by one in six people, who have a 1.5-fold greater risk of coronary diseases. Kamstrup et al. explained 21% and 27% of the total interindividual variation in Lp(a) levels in two independent samples of the general population according to copy-number variations of the kringle IV type 2 (KIV-2) repeat [5]. Moreover, they showed that the risk of myocardial infarction (MI) increased with decreasing numbers of KIV-2 repeats, and is associated with high Lp(a) levels. The largest study to date, involving 460506 patients with over 5.1 million person years of follow-up time in primary and secondary prevention, showed that Lp(a) predicts atherosclerotic cardiovascular disease with a linear gradient across the distribution of Lp(a) [6]. Similar results were found in 3359 patients with previous coronary artery disease (CAD) treated with statins and with Lp(a) levels higher than 700 mg/L [7], which is almost identical to our patients.

The first detectable functional change in the arterial wall that is indicative of the atherosclerotic process is decreased endothelial function. This can now be measured noninvasively using high-resolution ultrasound measures of impaired flow-mediated dilatation (FMD) of the brachial artery [8]. This is followed by morphological changes to the arterial wall that can be measured as increased intima media thickness in the carotid artery (c-IMT) [9]. It is known that Lp(a) levels are an independent predictor of decreased FMD and increased c-IMT in healthy subjects [8]. In children with heterozygous familiar hypercholesterolemia and increased Lp(a) levels, FMD is decreased compared to healthy children [10,11], while Lp(a) has no influence on c-IMT as a measure of morphological properties [9]. Pulse wave velocity (PWV) is another functional change in the arterial wall, and it is independently associated with Lp(a) levels in elderly patients with type 2 diabetes mellitus [12] or arterial hypertension [13]. However, there are no data available for patients after MI who are receiving optimal lipid-lowering treatments and have very high Lp(a) levels. FMD was shown to be an independent predictor of future cardiovascular events in apparently healthy people [14] and in people with pre-existing CAD [15]. However, in patients with or without pre-existing cardiovascular disease, c-IMT was shown to be an independent predictor of future cardiovascular events [16].

Based on these findings, the focus of the current study was to evaluate the genetic variability of the *LPA* gene in the specific group of patients with high risk of reoccurring CAD and very high Lp(a) levels. Our aim was to determine whether for these patients the two *LPA* SNPs (rs10455872, rs3798220) and the KIV-2 repeats are associated with increased Lp(a) levels and with the functional and morphological characteristics of the arterial wall. The novelty of this study is that we evaluated these associations in a specific group of patients, i.e., those who were stable following MI and showed very high Lp(a) levels.

## 2. Materials and Methods

### 2.1. Patients

Patients aged between 18 and 65 years with clinically stable CAD of at least 6 months after MI were eligible for inclusion in the study. Only patients who had an MI before the age of 55 were included in the study. Moreover, the patients included in this study showed serum Lp(a) levels of 1000 mg/L, irrespective of LDL cholesterol levels, or showed serum Lp(a) levels >600 mg/L and LDL cholesterol >2.6 mmol/L. All of the patients had been prescribed β blockers and antiplatelet drugs: they were all taking angiotensin-converting enzyme inhibitors/angiotensin receptor blockers, and statins at the highest tolerated doses. Their therapies had not been changed for at least 8 weeks before entering the study.

The main exclusion criteria were elevated liver transaminases, by more than three times above the normal levels, severe renal impairment and serum creatinine >200 mmol/L, or history of acute illness in the previous 6 weeks. All of the procedures performed in this study that involved human participants were carried out in accordance with the ethical guidelines of the 1964 Declaration of Helsinki. Approval for this study was obtained from the National Medical Ethics Committee of the Republic of Slovenia (reference number: KME 0120-357/2018/8). Written informed consent was obtained from all of the patients prior to inclusion in the study.

### 2.2. Clinical Examination

The systolic and diastolic blood pressures were measured in the sitting position after a minimum of 10 min of rest. The mean of three measurements was determined. Anthropometric parameters were determined, and body mass index was calculated (weight in kilograms divided by square of height in meters).

### 2.3. Biochemical Analysis

The blood for laboratory analysis was collected in the morning after 12 h overnight fasting. Samples were drawn from the antecubital vein into vacuum 5 mL tubes containing clot activator (Vacutube, LT Burnik, Slovenia). Serum was obtained by 15 min centrifugation at 2000× *g*. Total cholesterol, high-density lipoprotein (HDL) cholesterol, triglycerides, and apolipoproteins A1 and B were determined in the fresh serum by standard colorimetric or immunologic assays on an automated biochemistry analyser (Fusion 5.1; Ortho-Clinical Diagnostics, Raritan, NJ, USA). Lp(a) was determined on the same biochemistry analyser using the Denka reagent (Randox, Crumlin, UK), which contains apo(a) isoform-insensitive antibodies, and therefore showed minimal apo(a) size-related bias. LDL cholesterol was calculated according to the Friedewald formula [17].

### 2.4. Genetic Analysis

Genetic analysis was performed at the Institute for Biochemistry and Molecular Genetics, Faculty of Medicine, at the University of Ljubljana (Slovenia). Genomic DNA was extracted from venous blood samples using FlexiGene DNA kit 250 (Qiagen, Hilden, Germany), following the manufacturer instructions. The *LPA* genotyping and analysis of *LPA* kringel repeats was performed with TaqMan Universal PCR Master Mix and the QuantStudio 7 Flex real-time PCR system (all Applied Biosystems, Foster City, CA, USA). The rs10455872 and rs3798220 *LPA* polymorphisms were analysed using TaqMan SNP genotyping assays (C_30016089_10, C_25930271_10; Applied Biosystems, Foster City, CA, USA). Ten percent of the samples were reanalysed to monitor the quality of the genotyping. The *LPA* kringel repeats were analysed in triplicate using multiplex qPCR with a custom TaqMan expression assay for exon 5 of the *LPA* gene, and TaqMan Copy Number Reference Assay for the RNAseP gene that was used as a single copy reference gene (both Applied Biosystems, Foster City, CA, USA). If there were any discrepancies of >0.25 in the Ct values in replicates for either assay in individual samples, the sample was reanalysed. The relative number of KIV-2 repeats representing average value of repeats on both alleles in the individual patients was determined using the CopyCaller v2.1 software (Applied Biosystems, Foster, CA, USA).

### 2.5. Ultrasound Measurements

#### 2.5.1. Brachial Artery Flow-Mediated Dilation

Endothelial function was assessed using brachial artery FMD, according to the guidelines [18]. The measurements for each patient were performed at the same time of day, after a 10 min rest. The patients rested in a supine position with the right arm extended, immobilised with foam, and supported at an angle of approximately 80° from the torso. Blood pressure was recorded on the contralateral arm with an automated sphygmomanometer (OSZ Digital Blood Pressure System, Welch Allyn Speidel & Keller, Skaneateles Falls, NY, USA). Another blood pressure cuff was placed around the right forearm. Brachial artery diameter was visualised 5 cm to 10 cm above the antecubital fossa. The echo-machine continuously tracked and recorded the brachial artery diameter. After 1 min of measurements of baseline brachial artery diameter, the forearm blood pressure cuff was inflated to 50 mmHg above systolic pressure for 4 min, to produce arterial occlusion. After the occlusion period, the cuff was rapidly deflated, which induced reactive hyperaemia, and the brachial artery diameter was recorded for 3 min. At the end of this measurement, the machine automatically provided the baseline and maximal brachial diameter and FMD (as the percentage change from baseline diameter of the brachial artery during reactive hyperaemia). All of the images were recorded and saved onto the external hard drive.

#### 2.5.2. Arterial Stiffness

All of the measurements for arterial stiffness were performed on the right common carotid artery using a linear vascular probe (working frequency, 5–13 MHz), as described in [19]. Testing was performed with the patients lying comfortably in a supine position with a head elevation of around 45° and a side tilt of 30° to the left, in a quiet room with an air temperature of 22 °C to 24 °C. The ultrasound machine (Aloka prosound α7; Hitachi Aloka Medical, Ltd., Tokyo, Japan) was also equipped with special software with an integrated high-resolution eTracking system (Hitachi Aloka, Wallingford, CT, USA) for automatic determination of the stiffness parameters of the common carotid artery through analysis of the pulse wave. The echo-tracker cursor-pair was placed onto the anterior and posterior walls of the common carotid artery, approximately 1 cm to 2 cm proximal to the carotid bulb. Pressure waveforms were noninvasively obtained using arterial diameter change waveforms automatically calibrated based on the systolic and diastolic blood pressures measured, as described above. The carotid artery local stiffness (β stiffness) and the local PWVs were automatically calculated as means of six beats. The measurements were repeated six times, and the means were calculated.

#### 2.5.3. Intima Media Thickness

All of the measurements were performed for both sides of the common carotid artery and the internal carotid artery, according to the guidelines [20]. The plaques in the bulbs were given descriptively, as whether or not they were present. The presence of plaques meant an intima–media thickness of >1.1 cm. Testing was performed with patients in a supine position and under the same conditions as describe above for the Vivid E95 ultrasound machine. Measurements were performed on three segments including the 1 cm of the internal carotid artery, carotid bifurcation and 1 cm of the distal common carotid artery. The thickness of the intima media in the marked part of the intima media of the carotid artery was automatically calculated using the EchoPac 2.0 program, as means ± standard deviation.

### 2.6. Statistical Analysis

The Hardy–Weinberg equilibrium was assessed using chi-squared tests. Pairwise linkage disequilibrium (measured as D’ and r2) between the two SNPs was calculated using the EMLD software (https://cge.mdanderson.org/~qhuang/Software/pub.htm, accessed on 28 August 2021). Haplotype frequencies were calculated using the PHASE software [4]. Kolmogorov–Smirnov tests were used to define variables showing normal distributions, with these data expressed as means ± standard deviations. The non-normally distributed variables are expressed as medians and range (lower and upper quartiles). A general linear model using age and body mass index as a covariate was used to determine the relationship between genotype and haplotype groups and Lp(a) levels. The differences between genotype and haplotype groups were calculated with one-way ANOVA. For rs3798220 and the AC haplotype, where only one patient had the CC genotype and two had the AC haplotype, Student’s *t*-tests were used. Pearson correlation analysis was performed to determine the correlation of KIV2 repeats with biochemical and ultrasound parameters. Statistical analysis was performed using IBM SPSS Statistics for Windows, (Version 25.0. Armonk, NY, USA: IBM Corp.) and GraphPad Prism version 6 for Windows (GraphPad Software, San Diego, CA, USA; www.graphpad.com, accessed on 28 August 2021). GPower was used to perform the power calculations [21]. *p* values < 0.05 were considered as statistically significant.

## 3. Results

### 3.1. Patient Characteristics

A total of 70 patients were included in the study. The detailed characteristics of the patients are given in Table 1. All of the patients presented with clinically stable CAD of at least 6 months after MI, with a mean age at first coronary event of 45 years. The majority of the patients were male, with median Lp(a) level of 1483 mg/L. All of the patients had their blood pressure well-controlled. Overall, 11.4% were diabetics, again with well-controlled disease, and 5.7% were smokers. Although all of the patients were receiving statins at the highest tolerated dose without/with ezetimibe, their LDL cholesterol was not within target levels according to recent guidelines [22]. Other lipid parameters such as total cholesterol, HDL cholesterol and triglycerides as well as apolipoprotein B and A1 were not remarkably different from patients after MI with normal Lp(a) values. There were no significant differences in age, sex and body mass index between the genotype and haplotype groups.

### 3.2. Genotype and Haplotype Frequencies and KIV2 Repeats

Samples from all of the patients were genotyped for the two SNPs: rs10455872 with the intron variant A>G and with minor allele frequency 0.02216 [23], and rs3798220 with the missense variant T>C and with minor allele frequency 0.05132 [23]. The genotype and haplotype frequencies are given in Table 2. The haplotypes were defined as A>G for rs10455872 and T>C for rs3798220, and were as follows: AT (58.7%), AC (16.3%), GT (24.9%) and GC (0.09%). As the design of the study was to include patients with CAD with very high Lp(a) levels, the power of the study was calculated post hoc and showed 41% and 60% for rs10455872 and rs3798220, respectively. The frequencies of the minor allele obtained in our group of patients were 0.071 and 0.014 for rs10455872 and rs3798220, respectively (Table 2). Hence, due to relatively low number of patients in our group, only one patient with CC genotype of rs3798220 and two AC haplotypes was found in our study. However, these data are in accordance with the allele frequency for the Caucasian population as shown in the Database of Single-Nucleotide Polymorphisms (dbSNP), National Center for Biotechnology Information, U.S. National Library of Medicine. Both polymorphisms were in Hardy–Weinberg equilibrium (*p* = 0.75 and 0.84, rs10455872 and rs3798220, respectively). Moderate linkage disequilibrium (D’ = 0.999; r^2^ = 0.0655) was detected for both SNPs in this group of patients.

All of the patients also had relative KIV-2 copy number determined. Genotyping revealed a range in relative number of KIV-2 repeats of 5.8 to 13.5 (mean 9.9). In patients with no variant alleles, the Lp(a) levels were 1487 mg/L; for patients with one variant allele, 1586 mg/L; and for patients with two variant alleles, 1611 mg/L; however, the differences here were not statistically significant (*p* = 0.853).

### 3.3. Association between LPA Genotypes and Haplotypes, and Biochemical Parameters

**Figure 1:**
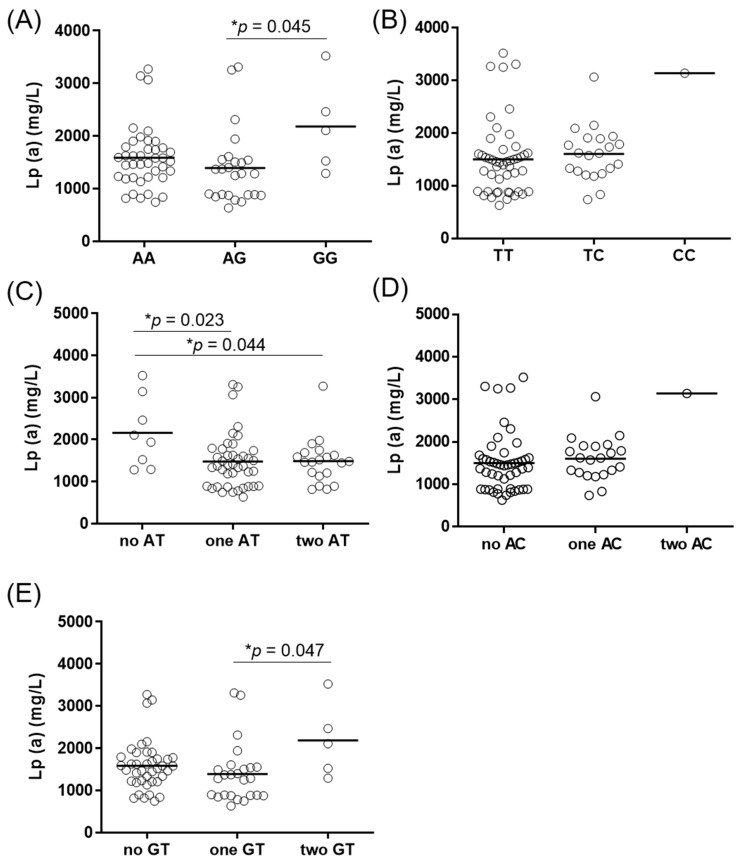
Lipoprotein(a) levels in *LPA* genotypes and haplotypes. The difference in Lp(a) serum levels between the rs10455872 genotype groups (**A**) and the rs3798220 genotype groups (**B**), and between their haplotypes (**C**–**E**). Analysis for rs3798220 and haplotype AC was performed using Student’s *t*-tests; the other p values were obtained using one-way ANOVA with Bonferroni post hoc analysis. * *p* < 0.05.

The Lp(a) levels in all of the genotype and haplotype groups are given in Figure 1. Genotype groups of rs10455872 showed statistically significant differences in Lp(a) levels (Figure 1a). Patients with the GG genotype of rs10455872 showed significantly higher Lp(a) levels in comparison to the AG genotype (median 2180 mg/L versus 1391 mg/L, *p* = 0.045). There were no statistical differences in Lp(a) levels for rs3798220 (Figure 1b). The general linear model showed that rs10455872 explained 6% of the variance in Lp(a) levels (*p* = 0.030), while rs3798220 explained 2.9%; however, this did not reach statistical significance (*p* = 0.082). Similarly, when both SNPs were included in the general linear model, the result of 10.4% of the variance in Lp(a) levels did not reach statistical significance (*p* = 0.702).

Haplotypes AT (Figure 1c) and GT (Figure 1e) showed statistically significant associations with Lp(a) levels, while no difference in Lp(a) levels was observed for the haplotype AC (Figure 1d). Patients with no AT haplotype were shown to have significantly higher Lp(a) levels in comparison with carriers of one AT haplotype (2158 mg/L versus 1478 mg/L, *p* = 0.023) or two AT haplotypes (2158 mg/L versus 1487 mg/L, *p* = 0.044). There was only one patient with both AC haplotypes in this study (Figure 1d), who had high Lp(a) levels (3143 mg/L). Thus, no statistical analysis was performed in comparison with those with no (1504 mg/L) or one (1608 mg/L) AC haplotype. Similarly, patients with both GT haplotypes had higher Lp(a) levels (2180 mg/L) in comparison with those with one (1391 mg/L, *p* = 0.045) GT haplotype. The difference in Lp(a) levels between those with both GT haplotypes and none (1586 mg/L) was not significant (*p* = 0.170). These data show that GT might be a risk haplotype for higher Lp(a) levels, while the presence of one or two copies of the AT haplotype appears to be protective from high Lp(a) levels, even in patients with very high Lp(a) levels.

On the other hand, no differences were seen between genotypes and haplotypes for total, LDL and HDL cholesterol, triglycerides, apolipoprotein A1 and apolipoprotein B (Table 3). Our data also suggest that AC haplotype might be a risk haplotype for high Lp(a) values, however we need to emphasise that only one patient with two copies of AC haplotype was included in our study.

**Figure 2:**
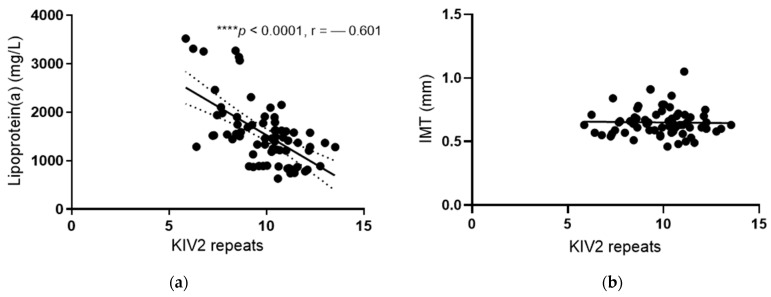
Correlation of Lp(a) levels and IMT with KIV2 repeats. Pearson correlation analysis was performed to determine the correlation of KIV2 repeats with Lp(a) levels (**a**) and IMT (**b**), respectively. **** *p* < 0.0001.

Lp(a) levels were inversely correlated with the number of KIV-2 repeats (r = −0.601; *p* < 0.0001) (Figure 2a). The number of KIV-2 repeats explained 36% of all of the variations in the population. In patients with KIV-2 repeats below the median (10.2), the Lp(a) levels were significantly higher compared with those with KIV-2 repeats above the median (1690 mg/L versus 1282 mg/L, *p* < 0.0001, Table 4). There were no differences in the levels of other lipids between these two groups of patients with KIV-2 repeats below and above the median (Table 4).

### 3.4. Association between LPA Genotypes and Haplotypes and Ultrasound Measurements

**Figure 3:**
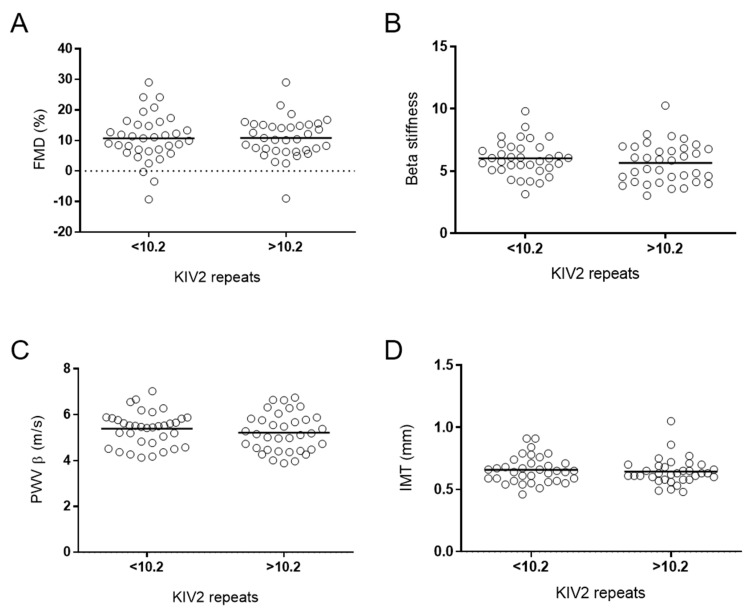
Morphological and functional parameters of arterial wall and KIV2 repeats. Morphological and functional parameters were compared between two groups of KIV2 repeats based on the median of KIV2 repeats, i.e., 10.2, using Student’s *t*-tests. (**A**) FMD, flow-mediated dilatation; (**B**) beta stiffness; (**D**) IMT, intima–media thickness; (**C**) PWV β, pulse wave velocity β.

There were no statistically significant differences in the functional and morphological arterial wall properties between the different genotypes and haplotypes (Table 5), or between groups with numbers of KIV-2 below and above the median (Figure 3). There were no correlations between IMT and the number of KIV-2 repeats (Figure 2b).

## 4. Discussion

To the best of our knowledge, this is the first study that has examined the association of two *LPA* SNPs, namely rs10455872 and rs3798220, and of variations in KIV-2 repeat numbers with Lp(a) levels and functional and morphological properties of the arterial wall in patients after MI and with very high Lp(a) levels. These data show that in this group of patients, the two SNPs and the KIV-2 repeat variations are associated with Lp(a) levels, but not with the functional and morphological properties in the arterial wall.

Clarke et al. were the first to identify the association of *LPA* SNPs rs3798220 and rs10455872 with increased Lp(a) levels and increased risk of CAD, when they investigated a population of 3145 patients with pre-existing CAD and 3352 control subjects [4]. Among the 33 SNPs that were associated with both Lp(a) levels and occurrence of future cardiovascular events, these two SNPs explained 8% and 25% of the variation in the Lp(a) levels, as rs3798220 and rs10455872, respectively. Together, both of these SNPs explained 36% of the variation in the Lp(a) levels. In the present study, a significant relationship was shown only for rs10455872, which explained 6% of the variance in Lp(a) levels (*p* = 0.030). The effects of rs3798220 and both of the SNPs on Lp(a) were 2.9% and 10.4%; however, these were not statistically significant. Clarke et al. demonstrated that those two SNPs also define the highest risk for future cardiovascular events, namely 1.92 and 1.70, for rs3798220 and rs10455872, respectively [4]. The main difference between the study by Clarke et al. and the present study is that the patient Lp(a) levels were 4.5-fold higher here than in their patients (1483 mg/L versus 330 mg/L). Moreover, all of the patients in the present study had CAD. There were also differences in the genotype and haplotype distributions between the present patient cohort and that of Clarke et al. (Table 2), whereby Clarke et al. showed 88.5% for the AT allele, 9.3% for the GT allele, and 2.3% for the AC allele. In the present study, the frequency was similar for the AT allele (88.6%), but higher for the presence of one or two copies of the GT allele (42.8%) and the AC allele (31.4%). In the present study, 28.6% of the patients had two AT alleles; i.e., the protective alleles, which means that they had no variations in either of the SNPs. Clarke et al. showed that patients with increasing numbers of variant alleles had significantly greater Lp(a) levels [4].

Regarding future cardiovascular events, Clarke et al. showed that increasing numbers of variant alleles were associated with increased risk of future cardiovascular events [4]. As the present study is cross-sectional, we cannot calculate the risk of future cardiovascular events. In patients with one variant allele, the risk was almost doubled (1.73), while in patients with two variants alleles, it was almost five times higher (4.87). In a study that included two general populations in Denmark with 69,764 patients, they showed that increased Lp(a) levels are an independent risk factor for cardiovascular and all-cause mortality [24]. In that study, Lp(a) levels were more connected with low numbers of KIV-2 repeats than with *LPA* rs10455872. They showed that KIV-2 repeats are more predictive than rs10455872 for Lp(a) levels, and for cardiovascular and all-cause mortality [24]. Similarly, in the present study, KIV-2 repeats explained 36% of the variability of Lp(a), and rs10455872 explained only 6%, while rs3798220 did not show any significant associations with Lp(a) levels. Another study from the same group that included 2461 patients with ischemic heart disease showed that doubling of Lp(a) levels increased the hazard ratio for MI by 22% [5]. The Lp(a) levels were mainly dependent on the number of KIV-2 repeats. The KIV-2 repeat variants explained 21% and 27% of the total Lp(a) variation [5]. In the present study, the number of KIV2 repeats was lower compared to previous studies [5,24]. This is expected as we have only included patients with very high Lp(a) levels.

Acute coronary events are predominantly due to ruptures of atherosclerotic plaques and the subsequent arterial thrombosis, which is dependent on the coagulation/fibrinolysis equilibrium [25]. The number of KIV-2 repeats define the apo(a) structure of the Lp(a), which is similar overall to plasminogen. Due to these similarities, apo(a) either binds to fibrin or forms a complex with fibrin, plasminogen and tissue plasminogen activator. The result of these two actions is decreased fibrinolysis, and thus increased risk of thrombotic events [26].

Raitakari et al. reported that in healthy patients, Lp(a) was not associated with either endothelial function or c-IMT, while LDL cholesterol was an independent predictor for both of these [8]. In that study, the patients received no cardiovascular therapy, and had substantially lower Lp(a) levels [6], with LDL cholesterol about 30% higher than in the present study. On the other hand, in children with heterozygous familial hypercholesterolemia and Lp(a) levels approximately a third of those of our patients, Lp(a) emerged as an independent predictor of FMD, but not of c-IMT or PWV [11]. In another study, the children had Lp(a) levels in the same range as in the previous study and moderately increased LDL cholesterol; here, Lp(a) was independently associated with FMD [10]. In contrast to all of these studies [8,10,11], in the present study, all of the patients were receiving statins and angiotensin-converting enzyme inhibitors or angiotensin receptor blockers, which have all been shown to have favourable effects on endothelial function [27,28] and c-IMT [29,30]. In patients with heterozygous familiar hypercholesterolemia treated with statins, there were no differences in c-IMT, and prevalence of carotid artery plaques in the subgroups with high versus low (<300 mg/L) Lp(a) levels [31].

Although arterial stiffness (measured as PWV) has been shown to be an independent predictor for a first cardiovascular event in the general population [32], there are very limited data available regarding the influence of Lp(a) on arterial stiffness [12,13,33]. Patients with CAD were only included in the study of Morishita et al., although they had Lp(a) levels that were lower than in the present study [13]. PWV correlated not only with Lp(a), but even more significantly with the oxidised form of Lp(a), which was shown to even potentiate the atherosclerotic effects of Lp(a), through attenuated plasminogen activator inhibitor 1 production in endothelial cells [34] and endothelial function [35].

Functional and structural properties to the arterial wall are associated not only with the presence of risk factors, but they are the products of the duration and intensity of particular risk factors. In these patients with very high Lp(a) levels that were determined genetically and were present from early childhood onwards, we can assume that functional and morphological changes occurred as early as in childhood. Vice versa, improvements in both FMD and c-IMT correlate with the product of the duration and intensity of the treatment. As the present patients were treated with statins on average for a duration of 10 years, which are known to decrease LDL cholesterol but not to have any deleterious effects on Lp(a) [36], we can assume that the improvements in FMD, c-IMT and PWV were not as good as in patients with increased LDL cholesterol and normal Lp(a) levels. This might be why there were no correlations between functional and morphological properties of the arterial wall and LDL cholesterol or Lp(a).

The limitations of the present study include the particularly low number of patients. Assuming an alpha level of 0.05, an additive inheritance model, an assumed prevalence of disease of 30%, a power of the study of 80%, minor allele frequencies between 1% (rs3798220) and 7% (rs10455872), and genetic relative risks between 1.1 and 1.3 (which can be expected in genetic association studies on complex diseases such as CAD), the number of patients in our study should be larger. The absence of the control group is also a limitation of the current study. Since this is a cross-sectional clinical study, the results are merely associations between the studied genetic variants and clinical parameters. To determine causality between these genetic variants and Lp(a) levels, further functional tests are needed. However, our primary aim was to focus on a specific group of patients; i.e., those with very high Lp(a) levels and MI before the age of 55 years.

## 5. Conclusions

In conclusion, the present study shows that the LPA genotypes rs3798220 and rs10455872 and their haplotypes are associated with Lp(a) levels in patients in a stable phase after an acute coronary event and with very high Lp(a) levels. On the other hand, there was no association between the genotypes nor haplotypes and the functional and morphological properties of the arterial wall. There was also no correlation between Lp(a) levels and the functional and morphological properties. This might be because these patients had long-lasting and greatly increased Lp(a) levels, which had the deleterious effect of increased LDL-cholesterol levels, and the beneficial effects of statins treatment. To clarify this question, further studies that include larger numbers of patients that differentiate only in terms of the Lp(a) levels would be needed.

## Figures and Tables

**Table 1:** Patient characteristics.

Parameter	Value (n = 70)
Male/female	61/9
Age (years)	51.2 ± 8.5
Age at first acute coronary event (years)	45.1 ± 6.6
Body mass index (kg/m^2^)	28.5 ± 4.0
Systolic blood pressure (mm Hg)	128 ± 13
Diastolic blood pressure (mm Hg)	77 ± 8
Smokers (%)	5.7
Diabetes mellitus (%)	11.4
Total cholesterol (mmol/L)	4.38 ± 0.83
HDL cholesterol (mmol/L)	1.14 ± 0.23
LDL cholesterol (mmol/L)	2.40 ± 0.77
Triglycerides (mmol/L)	1.84 ± 0.94
Lipoprotein(a) (mg/L)	1483 (1171–1780)
Apolipoprotein B (g/L)	0.85 ± 0.21
Apolipoprotein A1 (g/L)	1.30 ± 0.16

Data are means ± standard deviation for data with normal distribution, and median (lower–upper quartiles) for data not distributed normally.

**Table 2:** Genotype/haplotype frequency distributions.

SNP/Haplotype	n (%)
**rs10455872 A>G**	
A/A	40 (57.1)
A/G	25 (35.7)
G/G	5 (7.1)
**rs3798220 T>C**	
T/T	48 (68.1)
T/C	21 (30.0)
C/C	1 (1.4)
**AT**	
no AT	8 (11.4)
one AT	42 (60.0)
two AT	20 (28.6)
**AC**	
no AC	48 (68.6)
one AC	21 (30.0)
two AC	1 (1.4)
**GT**	
no GT	40 (57.1)
one GT	25 (35.7)
two GT	5 (7.1)

**Table 3:** Lipid profiles for the different *LPA* genotype and haplotype subgroups.

Parameter	Subgroup			*p* Value
**rs10455872**	**A/A**	**A/G**	**G/G**	
Total cholesterol (mmol/L)	4.2 ± 0.8	4.2 ± 0.8	4.7 ± 1.3	0.402
LDL (mmol/L)	2.3 ± 0.7	2.3 ± 0.7	2.7 ± 1.4	0.496
HDL (mmol/L)	1.1 ± 0.2	1.2 ± 0.3	1.3 ± 0.1	0.237
Triglycerides (mmol/L)	1.7 ± 0.9	1.6 ± 0.8	1.7 ± 0.7	0.927
Apolipoprotein A1 (g/L)	1.3 ± 0.1	1.4 ± 0.2	1.4 ± 0.1	0.132
Apolipoprotein B (g/L)	0.8 ± 0.2	0.8 ± 0.2	0.8 ± 0.2	0.869
**rs3798220**	**T/T**	**T/C**	**C/C**	
Total cholesterol (mmol/L)	4.2 ± 0.9	4.4 ± 0.7	4.1	0.386
LDL (mmol/L)	2.3 ± 0.8	2.4 ± 0.6	2.3	0.404
HDL (mmol/L)	1.1 ± 0.2	1.2 ± 0.3	1.1	0.663
Triglycerides (mmol/L)	1.7 ± 0.8	1.7 ± 1.0	1.4	0.910
Apolipoprotein A1 (g/L)	1.3 ± 0.2	1.3 ± 0.2	1.2	0.923
Apolipoprotein B (g/L)	0.8 ± 0.2	0.8 ± 0.2	0.9	0.581
**Haplotype AT**	**no AT**	**one AT**	**two AT**	
Total cholesterol (mmol/L)	4.5 ± 1.1	4.3 ± 0.8	4.0 ± 0.9	0.229
LDL (mmol/L)	2.5 ± 1.1	2.4 ± 0.7	2.1 ± 0.8	0.329
HDL (mmol/L)	1.3 ± 0.2	1.2 ± 0.3	1.1 ± 0.2	0.116
Triglycerides (mmol/L)	1.5 ± 0.6	1.7 ± 0.9	1.7 ± 0.9	0.799
Apolipoprotein A1 (g/L)	1.4 ± 0.2	1.3 ± 0.2	1.3 ± 0.1	0.257
Apolipoprotein B (g/L)	0.8 ± 0.2	0.8 ± 0.2	0.8 ± 0.2	0.960
**Haplotype AC**	**no AC**	**one AC**	**two AC**	
Total cholesterol (mmol/L)	4.2 ± 0.9	4.4 ± 0.7	4.1	0.710
LDL (mmol/L)	2.3 ± 0.8	2.4 ± 0.6	2.3	0.750
HDL (mmol/L)	1.2 ± 0.2	1.2 ± 0.3	1.1	0.889
Triglycerides (mmol/L)	1.7 ± 0.8	1.7 ± 1.0	1.4	0.933
Apolipoprotein A1 (g/L)	1.3 ± 0.2	1.3 ± 0.2	1.2	0.781
Apolipoprotein B (g/L)	0.8 ± 0.2	0.8 ± 0.2	0.9	0.794
**Haplotype GT**	**no GT**	**one GT**	**two GT**	
Total cholesterol (mmol/L)	4.2 ± 0.8	4.2 ± 0.8	4.7 ± 1.3	0.402
LDL (mmol/L)	2.3 ± 0.7	2.3 ± 0.7	2.7 ± 1.4	0.496
HDL (mmol/L)	1.1 ± 0.2	1.2 ± 0.3	1.3 ± 0.1	0.237
Triglycerides (mmol/L)	1.7 ± 0.9	1.6 ± 0.8	1.7 ± 0.7	0.927
Apolipoprotein A1 (g/L)	1.3 ± 0.1	1.4 ± 0.2	1.4 ± 0.1	0.152
Apolipoprotein B (g/L)	0.8 ± 0.2	0.8 ± 0.2	0.8 ± 0.2	0.869

Data are means ± standard deviations. *p* values for rs3798220 and haplotype AC were obtained using Student’s *t* tests. Other *p* values were obtained using one-way ANOVA. LDL, low-density lipoprotein; HDL, high-density lipoprotein.

**Table 4:** Lipids in the KIV-2 repeats groups.

Parameter	KIV-2 Repeat Groups		*p* Value
	<10.2	>10.2	
Total cholesterol (mmol/L)	4.2 ± 0.9	4.2 ± 0.8	0.936
LDL (mmol/L)	2.3 ± 0.9	2.3 ± 0.7	0.752
HDL (mmol/L)	1.2 ± 0.3	1.2 ± 0.3	0.951
Triglycerides (mmol/L)	1.6 ± 0.9	1.8 ± 0.8	0.379
Apolipoprotein A1(g/L)	1.3 ± 0.2	1.3 ± 0.2	0.905
Apolipoprotein B(g/L)	0.8 ± 0.2	0.8 ± 0.2	0.556
Lipoprotein(a) (mg/L)	1690 (1335–2105)	1282 (873–1579)	<0.0001

Data are means ± standard deviation. For Lp(a), data are median (lower–upper quartile). *p* values were obtained using Student’s *t* test. LDL, low-density lipoprotein; HDL, high-density lipoprotein.

**Table 5:** Functional and morphological properties of the arterial wall for the different *LPA* genotype and haplotype subgroups.

Parameter	Subgroup			*p* Value
**rs10455872**	**A/A**	**A/G**	**G/G**	
FMD (%)	10.6 ± 7.3	11.0 ± 7.6	11.0 ± 3.2	0.942
Beta stiffness	6.0 ± 1.5	5.7 ± 1.6	5.6 ± 1.0	0.678
PWV β (m/s)	5.4 ± 0.7	5.2 ± 0.9	5.3 ± 0.8	0.580
c-ITM (mm)	0.66 ± 0.11	0.63 ± 0.11	0.65 ± 0.11	0.601
**rs3798220**	**T/T**	**T/C**	**C/C**	
FMD (%)	10.4 ± 6.0	11.4 ± 9.3	14.8	0.640
Beta stiffness	5.7 ± 1.6	6.1 ± 1.4	5.5	0.386
PWV β (m/s)	5.2 ± 0.8	5.5 ± 0.8	5.2	0.145
c-ITM (mm)	0.65 ± 0.10	0.65 ± 0.12	0.68	0.947
**Haplotype AT**	**no AT**	**one AT**	**two AT**	
FMD (%)	14.5 ± 6.7	10.1 ± 7.6	10.8 ± 6.0	0.277
Beta stiffness	5.5 ± 0.9	5.9 ± 1.5	5.8 ± 1.6	0.796
PWV β (m/s)	5.2 ± 0.8	5.4 ± 0.8	5.2 ± 0.7	0.580
c-ITM (mm)	0.64 ± 0.09	0.64 ± 0.12	0.67 ± 0.09	0.691
**Haplotype AC**	**no AC**	**one AC**	**two AC**	
FMD (%)	10.4 ± 6.0	11.4 ± 9.3	14.8	0.733
Beta stiffness	5.7 ± 1.6	6.1 ± 1.4	5.5	0.666
PWV β (m/s)	5.2 ± 0.8	5.5 ± 0.8	5.2	0.341
c-ITM (mm)	0.65 ± 0.10	0.65 ± 0.12	0.68	0.956
**Haplotype GT**	**no GT**	**one GT**	**two GT**	
FMD (%)	10.6 ± 7.3	11.0 ± 7.6	11.0 ± 3.2	0.979
Beta stiffness	6.0 ± 1.5	5.7 ± 1.6	5.6 ± 1.0	0.678
PWV β (m/s)	5.4 ± 0.7	5.2 ± 0.9	5.3 ± 0.8	0.580
c-ITM (mm)	0.66 ± 0.11	0.63 ± 0.11	0.65 ± 0.11	0.601

Data are means ± standard deviations. *p* values for rs3798220 and haplotype AC were obtained using Student’s *t* tests. Other *p* values were obtained using one-way ANOVA. LDL, low-density lipoprotein; HDL, high-density lipoprotein.

## Data Availability

The data presented in this study are available on request from the corresponding author. The data are not publicly available due to protection of the privacy of personal data.

## Abbreviations

ApoA1	apolipoprotein A
ApoB	apolipoprotein B
CAD	coronary artery disease
c-IMT	intima–media thickness in the carotid artery
FMD	flow-mediated dilatation
HDL	high-density lipoprotein
LDL	low-density lipoprotein
Lp(a)	lipoprotein(a)
*LPA*	lipoprotein(a) gene
MI	myocardial infarction
SNP	single-nucleotide polymorphism
PWV	pulse wave velocity
